# Anæsthesia and Analgesia

**Published:** 1912-06

**Authors:** 


**Anæsthesia and Analgesia.** By J. D. MORTIMER, M.B. Lond., F.R.C.S. Eng. Pp. x, 276. London: University of London Press. 1911.

In this volume of London Practitioners' Manuals Dr. Mortimer has refrained from dealing with the historical or chemical aspects of his subject, and has successfully set himself the task of laying before his readers such details and rules of administration as may be of special value for the guidance of the anæsthetist in his ordinary practice. A work of this description does not readily lend itself to originality, and as a matter of fact, we fail to find in these pages much information which has not appeared under other authors.

In many instances the treatment of the subject suffers by being too highly condensed. For instance, in the section dealing with the administration of ether by the open method, no mention is made of any form of covering for the wire mask other than one composed of from eight to twelve layers of gauze. As this fabric is apt to give a quite unnecessary concentration of ether vapour, its promiscuous use is prone to lead to undesirable after-effects.

Chapter xii is devoted to spinal analgesia, and contains a clear "summary of conclusions," which terminate with the following warning: "That this (spinal) method should not be adopted without some definite reason." It seems a pity that the author did not here insert a table showing exactly in what particular conditions he considers general anæsthesia to be inadmissible, or to be fraught with graver risks than is spinal analgesia. Such a list would be extremely useful.

